# Impact of coronavirus disease 2019 (COVID-19) pandemic on attitude, behavior, and mental health of patients with rheumatic diseases

**DOI:** 10.1186/s43166-020-00045-y

**Published:** 2020-11-12

**Authors:** Marwa A. H. Hammad, Mervat Eissa, Ghada A. Dawa

**Affiliations:** 1 Rheumatology and Rehabilitation Department, Faculty of Medicine, Zagazig University, Zagazig, Egypt; 2 Rheumatology Department, Faculty of Medicine, Cairo University, Cairo, Egypt

**Keywords:** COVID-19, Rheumatic patients, Attitude, Behavior, Mental health

## Abstract

**Background:**

The coronavirus disease 2019 (COVID-19) pandemic has become a global health, social, and economic crisis. Healthcare professionals, patients, healthy individuals, and the whole community are under inevitable psychological pressure which may cause different psychological problems as fear, anxiety, depression, and insomnia. The aim was to assess the impact of the COVID19 pandemic on the attitude, behavior, and mental health of rheumatic patients and to compare them with healthy individuals. This is a case-control study, 360 participants were included and divided into a patient group composed of 180 patients with rheumatic diseases, and a control group composed of 180 healthy people. Data were collected via a self-administered structured questionnaire designed on Google forms. It was sent to participants via social networks and emails to different rheumatic patients and healthy individuals. Mental health was measured by the 5-item Brief Symptom Rating Scale (BSRS-5).

**Results:**

The mean age of cases and control were (35.05 ± 8.79 vs 34.56 ± 9.06) years. In comparing attitudes and behavior toward COVID 19, there was a statistically significant difference (*p* ≤ 0.05) between both groups regarding washing hands, going outdoors, wearing masks and gloves outdoors, and staying in their rooms. Patients depended mainly on telehealth more than usual where about 50% used either phone calls, internet or sent their relatives to their physicians; moreover, 20% did not contact their physicians at all the past few months. There was a statistically significant difference (*p* ≤ 0.05) between both groups regarding feeling angry/irritated, inferior and insomniac. The BSRS-5 total score and being defined as a psychiatric case (according to the BSRS-5 scale) also differed significantly between patients and controls. Systemic lupus erythematosus (SLE) patients showed more adherence to their medications and stayed mostly at home and they have higher BSRS scores.

**Conclusion:**

Patients with rheumatic diseases show comparable degrees of anxiety and depression to healthy individuals, but higher distress symptoms and panic in the form of anger, irritability, and insomnia. They have a significantly higher sense of inferiority and a higher total BSRS compared to controls. SLE patients show more adherence to their medications and stay mostly at home as a reflection of feeling more vulnerable. Moreover, they have higher degrees of psychological affection in the form of higher BSRS scores. Abandoning drug purchasing without medical prescription is necessary in Egypt to protect our patients from unnecessary drug shortages adding to their fear and anxiety. Mental health should be addressed in the same manner we deal with the infectious disease itself, being of no less importance. Mental health professionals, social workers, and support groups need to provide psychological support to vulnerable populations, including patients with rheumatic diseases. Rheumatologists should be aware of the need for psychiatric consultation for their patients whenever necessary.

## Background

The corona virus disease 2019 (COVID-19) is an infectious disease caused by the severe acute respiratory syndrome coronavirus 2 (SARS-CoV-2), which originated in Wuhan, China, in early December 2019 and declared a pandemic by the World Health Organization (WHO) on 11 March 2020 [1, 2]. Previous data from similar outbreaks, such as the Severe Acute Respiratory Syndrome [SARS], showed that the community suffered considerable anxiety, depression, and panic, resulting in a significant psychological impact [3–5].

The mortality of COVID-19 is even higher than previous epidemics inducing profound fear [6, 7]; mental health reflections can last longer and even have a higher frequency than the epidemic itself [8, 9]. Shortages of masks and health equipment, quarantine, lack of effective treatment, closure of schools and public places, boredom, fear of infection, or death could increase panic. Moreover, it can lead to a feeling of insecurity due to economic and social losses [10–14].

Patients with rheumatic diseases—those suffering from rheumatoid arthritis, systemic lupus, scleroderma, ankylosing spondylitis, Sjogren’s disease, inflammatory myositis, fibromyalgia, or others—are considered at greater risk both due to their chronic illnesses and use of immunosuppressive drugs [15]. They are also facing many issues- drug shortages, difficulties reaching clinics or hospitals, and hazards of physical inactivity staying at home. It is expected that these individuals with chronic diseases will be prone to higher levels of impaired psychology [16] since the COVID-19 tends to hit hardest those with multiple comorbidities [17]. The American College of Rheumatology [ACR] Issues COVID-19 Treatment Guidance for Rheumatic Disease Patients recommended the reduction of the number of health care encounters; laboratory monitoring less frequently, using telehealth, and increasing intervals between intravenous doses and they set the basis for when to start, stop, or reduce medications [18].

A recent public survey conducted in China about the psychological impact of the COVID-19 outbreak showed that about a third of participants experienced moderate to severe anxiety and about half experienced moderate to severe psychological impairment [19]. No previous researches studied the psychological impact of COVID-19 on patients with rheumatic diseases in Egypt. This present study represents the first mental health survey conducted in Egypt addressing this vulnerable population. From this point we aimed to assess the impact of [COVID-19] pandemic on the attitude, behavior, and mental health of patients with rheumatic disease and to compare them with healthy individuals.

## Methods

### The study design

This is an Egyptian case-control study, sample size was calculated based on findings from the pilot study prevalence among cases 17% and prevalence among control 40% case to control 1:1. A total of 360 participants were included consecutively in this study and divided into two groups: a patient group composed of 180 patients with rheumatic diseases and a control group composed of 180 healthy individuals. Rheumatic patients and healthy individuals had been recruited from 14 to 29 April 2020. Data were collected via a self-administered structured questionnaire that were delivered as a Google form in Arabic, which were sent to participants via social networks targeting rheumatic patients and healthy individuals, or via emails. The survey was translated into Arabic [mother tongue of Egyptians] and validated by the authors. It took about 5–10 min to be completed. The participants' information is kept anonymous. The translated form is available in the supplemental files. All control subjects were age, sex, socio-economically, and geographically matched with the patients [living allover Egypt those educated enough to use social media and internet]. The study complied with the Declaration of Helsinki, and informed online consent has been obtained from the subjects. Ethical approval was taken from Institutional Review Board IRB under number of (ZU-IRB #6096).

### The questionnaire

It was designed as a self-administered questionnaire with closed questions [yes/no questions, rating scale, and multiple-choice questions] and some open-ended questions. It was composed of 7 parts and a total of 37 questions.

1. *Description* of the survey, its aim, targeted population, and time needed to fill it.
2. *Participants’ characteristics*, including age, gender, nationality, residential area, smoking, infection with COVID19, defining his/her rheumatic disease, and previous lung affection.
3. *A question about the most reliable information source about COVID19* they relied on TV/radio, internet, social media groups, own physicians, friends, and family.
4. *Five questions about their attitude towards some protective measures* [such as staying at home, wearing protective masks, wearing gloves, washing hands with water and soap, eating a healthy diet and exercises help]; answers were “yes,” “no,” or “maybe”
5. *Five questions about their behavior* [going outdoors, wearing masks outdoors and wearing gloves outdoors, spending much time in the room away from the family] and frequency of washing hands]; graded as a Likert-type scale “never,” “a bit of the time/few times,” “most of the time”, and “always” based on the question.
6. *Eight questions for rheumatic patients only about*: contact with the physician, the means and causes of contact and their feeling afterward, their attitude towards immunosuppressive drugs.
7. *Six questions for mental health assessment for patients and controls*: Mental health was measured by the *5-item Brief Symptom Rating Scale [BSRS-5]* which was derived from the 50-item BSRS [20]. This effective screening instrument has satisfactory reliability and validity and is widely used to identify psychiatric impairment in medical settings and community samples [20–22]. The scale was translated and back-translated to Arabic language and a pilot study was conducted to test the applicability and the clarity of the scale then changes were made accordingly. It consisted of five symptom items, namely:
  Feeling tense or high-strung [anxiety];
  Feeling depressed or in a low mood [depression];
  Feeling easily annoyed or irritated [hostility];
  Feeling inferior to others [inferiority];
  Having trouble falling asleep [insomnia];

The score for each item ranges from 0 to 4 [0, not at all; 1, a little bit; 2, moderately; 3, quite a bit; and 4, extremely], based on a 5-point Likert-type scale, participants were asked to indicate how much discomfort they experienced that was caused by a particular symptom in the past week [including the current day]. The sum of the scores across the five items represented the total score of the BSRS-5 and ranged from 0 to 20. A total score on the BSRS-5 above 14 may indicate a severe mood disorder. Scores between 10 and 14 may indicate moderate mood disorders, and those between 6 and 9 could indicate mild mood disorders. A cut-off score of ≥ 6 was considered as a BRSR-5 defined psychiatric case [20].

### Statistical analysis

The collected data were statistically analyzed using the Statistical Package for Social Science software [SPSS] [Version 20.0. Armonk, NY: IBM Corp.]. Continuous variables with a normal distribution were reported as mean and standard deviation [SD]. Categorical variables were summarized as frequencies and percentages. Quantitative data were evaluated using Independent *t* test or one-way ANOVA, which were suitable for normally distributed data, while qualitative data were evaluated by chi-square test [*χ2*] or Fisher’s exact test. *p* values ≤ 0.05* and ≤ 0.001** were considered statistically significant and highly statistically significant, respectively.

## Results

Socio-demographic and personal characteristics of the studied groups showed that there was no statistically significant difference [*p* > 0.05] between both groups; ensuring their comparability. The mean age of cases and control groups were 35.05 ± 8.79 vs 34.56 ± 9.06 years with the highest proportion of them were females [78.3% vs 77.8%], non-smokers [83.3% vs 86.7%] and all of them were non-infected with COVID and about 48.9% vs 47.2% received their COVID information from social media. About 40% of the case group had RA and the majority of this group [78.3%] did not have previous lung affection (Table 1).

**Table 1:** Socio demographic and personal characteristics of the studied groups (*n* = 360)

Characteristics	Cases (***n*** = 180)	Controls (***n*** = 180)	Test	***p*** value
^**c d**^**Age (years)**				
Mean ± SD	35.05 ± 8.79	34.56 ± 9.06	^**a**^0.526	0.600
^**c d**^**Sex** no (%)				
Female	141 (78.3%)	140 (77.8%)	^**b**^0.016	0.899
Male	39 (21.7%)	40 (22.2%)		
**Smoking,** no (%)				
No	150 (83.3%)	156 (86.7%)	^**b**^0.784	0.376
Yes	30 (16.7%)	24 (13.3%)		
**Infected with COVID**, no (%)				
No	180 (100%)	180 (100%)	**-**	-
Yes	0.0 (0%)	0.0 (0%)		
**COVID information source**, no (%)				
TV/radio	39 (21.7%)	41(22.8%)	^**b**^5.926	0.205
Internet	42 (23.3%)	44 (24.4%)		
Social media	88 (48.9%)	85 (47.2%)		
My physician	9 (5%)	3 (1.7%)		
Friends and family	2 (1.1%)	7 (3.9%)		
**Rheumatological disease**, no (%)				
RA	72 (40%)	-	-	-
SLE	67 (37.2%)			
Rhupus	4 (2.2%)			
AS	9 (5%)			
Behcet	12 (6.7%)			
Sjogren	2 (1.1%)			
Vasculitis	1 (0.6%)			
Fibromyalgia	5 (2.8%)			
Polymyalgia	1 (0.6%)			
APA	1 (0.6%)			
Sarcoidosis	1 (0.6%)			
IBD	1 (0.6%)			
Scleroderma	1 (0.6%)			
DM	1 (0.6%)			
PSA	1 (0.6%)			
Mixed	1 (0.6%)			
**Lung affection**, no (%)				
No	141 (78.3%)	-	-	-
Yes	39 (21.7%)			

*COVID-19* coronavirus disease 2019, *TV* television, *RA* rheumatoid arthritis, *SLE* systemic lupus erythematosus, *AS* ankylosing spondylitis, *FMS* fibromyalgia, *APA* antiphospholipid syndrome, *IBD* inflammatory bowel syndrome, *DM* dermatomyositis, *PSA* psoriatic arthritis

^a^Independent *t* test

^b^Chi square test (*χ*^*2*^)

^c^ Significant between RA, SLE, others

^d^Significant between RA, SLE

On comparing attitude and behavior toward COVID-19 pandemic among the studied groups, no statistically significant difference [*p* > 0.05] was found between patients and the control group regarding their belief that staying at home protects them from infection, healthy diet and exercises help, and frequent washing hands, while there was a statistically significant difference [*p* ≤ 0.05] between both groups regarding their behavior regarding washing hands, going outdoors, wearing masks and gloves outdoors, and staying in their room (Table 2).

**Table 2:** Attitude and behaviour regarding COVID among the studied groups (*n* = 360)

Protective attitude/behavior	Cases (***n*** = 180)	Controls (***n*** = 180)	^**a**^ Test	***p*** value
	No (%)	No (%)		
**Staying at home helps**				
No	18 (10%)	9 (5%)	3.246	0.197
Yes	98 (54.4%)	103 (57.2%)		
Maybe	64 (35.6%)	68 (37.8%)		
**Wearing masks helps**				
No	^b^52 (28.9%)	20 (11.1%)	19.85	< 0.001**
Yes	57 (31.7%)	85 (47.2%)		
Maybe	71 (39.4%)	75 (41.7%)		
**Wearing gloves helps**				
No	^b^79 (43.9%)	44 (24.4%)	18.16	<0.001**
Yes	36 (20%)	64 (35.6%)		
Maybe	65 (36.1%)	72 (40%)		
**Washing hands helps**				
No	3 (1.7%)	1 (0.6%)	10.46	0.005*
Yes	^b^129 (71.7%)	154 (85.6%)		
Maybe	48 (26.7%)	25 (13.9%)		
^**c d**^**Healthy diet and exercises help**				
No	21 (11.7%)	16 (8.9%)	1.071	0.585
^c d^Yes	95 (52.8%)	103 (57.2%)		
^c^May be	64 (35.6%)	61 (33.9%)		
^**c**^**Going outdoors**				
^c^Never	48 (26.7%)	24 (13.3%)	12.64	0.002*
^c^Only for necessities	^b^113 (62.8%)	143 (79.4%)		
Go out freely	19 (10.6%)	13 (7.2%)		
**Wearing mask outdoors**				
Never	66 (36.7%)	54 (30%)	11.82	0.019*
Few times	^b^28 (15.6%)	44 (24.4%)		
Most of the time	31 (17.2%)	43 (23.9%)		
Always	22 (12.2%)	22 (12.2%)		
I do not go out	^b^33 (18.3%)	17 (9.4%)		
**Wearing gloves outdoors**				
Never	^b^90 (50%)	70 (38.9%)	19.33	0.001**
Few times	^b^21 (11.7%)	41 (22.8%)		
Most of the time	20 (11.1%)	27 (15%)		
Always	14 (7.8%)	25 (13.9%)		
I do not go out	35 (19.4%)	17 (9.4%)		
^**d**^**Staying in your room**				
^d^Move freely at home	133 (73.9%)	134 (74.4%)	10.11	0.018*
A bit of time	^b^19 (10.6%)	33 (18.3%)		
Most of time	26 (14.4%)	13 (7.2%)		
All of time	2 (1.1%)	0.0 (00%)		
**Frequency of washing hands**				
Less than 5 times	31 (17.2%)	26 (14.4%)	1.942	0.379
5 to 10 times	97 (53.9%)	90 (50%)		
More than 10 times	52 (28.9%)	64 (35.6%)		

^a^ Chi square test (*χ*^2^)

^b^Cell of significance

^c^Significant between RA, SLE, others

^d^Significant between RA, SLE. *P value is significant. **P value is highly significant

Mental health comparison among the studied groups showed that no statistically significant difference [*p* > 0.05] was found between the patients and control groups regarding anxiety and depression, while there was a statistically significant difference [*p* ≤ 0.05] between both groups regarding feeling angry/irritated, inferior, and insomniac. BSRS-5 total score and being defined as a psychiatric case according to BSRS-5 scale was significantly higher in patients (Table 3).

**Table 3:** Mental health comparison among the studied groups (*n* = 360)

Mental health	Cases (***n*** = 180), no (%)	Controls (***n*** = 180), no (%)	Test	***p*** value
**Anxiety**				
Not at all	17 (9.4%)	11 (6.1%)	^a^9.028	0.060
A little bit	34 (18.9%)	36 (20%)		
Moderately	54 (30%)	64 (35.6%)		
Quite a bit	49 (27.2%)	58 (32.2%)		
Extremely	26 (14.4%)	11 (6.1%)		
**Depression**				
Not at all	28 (15.6%)	37 (20.6%)	^a^6.405	0.171
A little bit	44 (24.4%)	45 (25%)		
Moderately	41 (22.8%)	50 (27.8%)		
Quite a bit	44 (24.4%)	36 (20%)		
Extremely	23 (12.8%)	12 (6.7%)		
**Angry/irritated**				
Not at all	4 (2.2%)	10 (5.6%)	^a^10.73	0.030*
A little bit	32 (17.8%)	44 (24.4%)		
Moderately	47 (26.1%)	58 (32.2%)		
Quite a bit	^c^59 (32.8%)	42 (23.3%)		
Extremely	38 (21.1%)	26 (14.4%)		
^**e**^**Inferiority**				
^e^Not at all	^c^63 (35%)	108 (60%)	^a^41.20	< 0.001**
A little bit	39 (21.7%)	43 (23.9%)		
Moderately	35 (19.4%)	22 (12.2%)		
Quite a bit	25 (13.9%)	3 (1.7%)		
Extremely	18 (10%)	4 (2.2%)		
**Insomnia**				
Not at all	20 (11.1%)	36 (20%)		
A little bit	^c^25 (13.9%)	60 (33.3%)		
Moderately	43 (23.9%)	43 (23.9%)		
Quite a bit	52 (28.9%)	27 (15%)		
Extremely	40 (22.2%)	14 (7.8%)	^a^39.41	< 0.001**
^**d e**^**BSRS-5 total score**				
Mean **±** SD	10.45 ± 4.22	8.16 ± 3.74	^b^5.466	< 0.001**
< 6 not psychiatric case	26 (14.4%)	45 (25%)	^a^6.334	0.012*
≥ 6 psychiatric case	154 (85.6%)	135 (75%)		
**Your fear about**				
Infection	26 (14.4%)	15 (8.3%)	^a^57.30	< 0.001**
Family	^c^92 (51.1%)	136 (75.6%)		
Quarantine	6 (3.3%)	15 (8.3%)		
Financial problems	14 (7.8%)	14 (7.8%)		
Medications	11 (6.1%)	0.0 (00%)		
Mixed fears	31 (17.2%)	0.0 (00%)		

^a^Chi square test (*χ*^2^)

^b^ Independent *t* test

^c^Cell of significance

^d^Significant between RA, SLE, others

^e^Significant between RA, SLE. *P value is significant. **P value is highly significant

Regarding patients, attitude, and behavior towards their illness, medications, and medical advice, the highest proportion of them answered that they felt at higher risk and they felt no change in their disease activity; however, they believed that fear may increase disease activity. They contacted their physicians less than usual, mainly using the internet. They mainly asked for medication advice and most of them still felt non-assured after communication. Most of them had no fears about drugs, did not reduce doses/stop drugs, and many experienced drug shortages (Table 4).

**Table 4:** Patients’ attitude and behavior regarding disease, medications and medical advice (*n* = 180)

Attitude/behavior	Value (***n*** = 180)	
	No	(%)
^**a b**^**Are you at risk**		
No	23	12.8
^a b^Yes	97	53.9
^a b^Maybe	60	33.3
**Current disease activity**		
Decreased	6	3.3
Did not change	131	72.8
Increased	43	23.9
**Disease activity may increase due to**		
Fear	92	51.1
Postponed follow-up visits	22	12.2
Reduced activity	17	9.4
Drug shortage	30	16.7
Drug incompliance	19	10.6
**Contacting your physician**		
Less than usual	80	44.4
As usual	44	24.4
More than usual	5	2.8
I did not	51	28.3
**Means of communication**		
Phone call	29	16.1
Clinic	50	27.8
Internet	63	35
Send someone	2	1.1
I did not	36	20
**Cause of communication**		
Disease activity or pain	44	24.4
COVID-19 symptoms	5	2.8
Drug shortage	24	13.3
Medication advice	51	28.3
Ask about COVID-19	6	3.3
I did not	50	27.8
^**a b**^**Feeling after communication**		
Feel more anxiety and fear	14	7.8
Feel the same	75	41.7
^a b^Feel less anxiety and fear	37	20.6
^a^I did not	54	30
**Fear of drugs (no/yes)**	108/72	60/40
^**a b**^**Stopped drugs (no/yes)**	148/32	82.2/17.8
^**a**^**Drug shortage (no/yes)**	57/123	31.7/68.3
**Reduce doses (no/yes)**	122/58	67.8/32.2
**Physician advice if you have COVID (no/yes)**	152/28	84.4/15.6

^a^Significant between RA, SLE, others

^b^Significant between RA, SLE. COVID-19; Coronavirus disease 2019

On assessing the relationship between different parameters and patients’ mental health degree, there was no statistically significant association [*p* > 0.05] between the degree of impaired mental health and all studied parameters except for “feeling at risk more than other” where there was a statistically significant association [*p* ≤ 0.05] where their feeling of “being at risk” was associated with a higher degree of impaired mental health [BSRS-5 ≥ 6] (Tables 5 and 6).

**Table 5:** Relation between different parameters and patients’ mental health (*n* = 180)

Parameters	BSRS-5 < 6 (***n*** = 26), no (%)	BSRS-5 ≥ 6 (***n*** = 154), no (%)	^**a**^ Test	***p*** value
**Age (years)** ≤ median (34) (*n* = 97)	12 (12.4%)	85 (87.6%)	0.732	0.392
**Female sex** (*n* = 141)	18 (12.8%)	123 (87.2%)	1.484	0.223
**Non-smoker** (*n* = 150)	22 (14.7%)	128 (85.3%)	Fisher	0.850
**COVID-19 information source**				
TV/radio (*n* = 39)	6 (15.4%)	33 (84.6%)	5.927	0.205
Internet (*n* = 42)	9 (21.4%)	33 (78.6%)		
Social media (*n* = 88)	10 (11.4%)	78 (88.6%)		
My physician (*n* = 9)	0.0 (00%)	9 (100%)		
Friends and family (*n* = 2)	1 (50%)	1 (50%)		
**Rheumatological disease**				
RA(*n* = 72)	16 (22.2%)	56 (77.8%)	5.887	0.053
SLE(*n* = 67)	6 (9%)	61 (91%)		
Others (*n* = 41)	4 (9.8%)	37 (90.2%)		
**No lung affection** (*n* = 141)	20 (14.2%)	121 (85.8%)	0.036	0.850
**Means of protection: Staying at home**				
No (*n* = 18)	5 (27.8%)	13 (72.2%)	2.895	0.235
Yes (*n* = 98)	13 (13.3%)	85 (86.7%)		
Maybe (*n* = 64)	8 (18.5%)	56 (87.5%)		
**Wearing masks**				
No (*n* = 52)	6 (11.5%)	46 (88.5%)	0.710	0.701
Yes (*n* = 57)	8 (14%)	49 (86%)		
Maybe (*n* = 71)	12 (16.9%)	59 (83.1%)		
**Wearing gloves**				
No (*n* = 79)	14 (17.7%)	65 (82.3%)	1.250	0.535
Yes (*n* = 36)	4 (11.1%)	32 (88.9%)		
May be (*n* = 65)	8 (12.3%)	57 (87.7%)		
**Washing hands**				
No (*n* = 3)	0.0 (00%)	3 (100%)	1.394	0.498
Yes (*n* = 129)	17 (13.2%)	112 (86.8%)		
Maybe (*n* = 48)	9 (18.8%)	39 (81.2%)		
**Healthy diet and exercises help**				
No (*n* = 21)	2 (9.5%)	19 (90.5%)	3.301	0.192
Yes (*n* = 95)	18 (18.9%)	77 (81.1%)		
Maybe (*n* = 64)	6 (9.4%)	58 (90.6%)		
**Going outdoors**				
Never (*n* = 48)	8 (16.7%)	40 (83.3%)	1.249	0.535
Only for necessities (*n* = 113)	14 (12.4%)	99 (87.6%)		
Go out freely (*n* = 19)	4 (21.1%)	15 (78.9%)		
**Wearing mask outdoors**				
Never (*n* = 66)	11 (16.7%)	55 (83.3%)	3.013	0.556
Few times (*n* = 28)	5 (17.9%)	23 (82.1%)		
Most of the time (*n* = 31)	2 (6.5%)	29 (93.5%)		
Always (*n* = 22)	2 (9.1%)	20 (90.9%)		
I do not go out (*n* = 33)	6 (18.2%)	27 (81.8%)		
**Wearing loves outdoors**				
Never (*n* = 90)	16 (17.8%)	74 (82.2%)	3.474	0.482
Few times (*n* = 21)	2 (9.5%)	19 (90.5%)		
Most of the time (*n* = 20)	1 (5%)	19 (95%)		
Always (*n* = 14)	1 (7.1%)	13 (92.9%)		
I do not go out (*n* = 35)	6 (17.1%)	29 (82.9%)		
**Staying in my room**				
Move freely at home (*n* = 133)	23 (17.3%)	110 (82.7%)	4.597	0.204
A bit of time (*n* = 19)	0.0 (00%)	19 (100%)		
Most of time (*n* = 26)	3 (11.5%)	23 (88.5%)		
All of time (*n* = 2)	0.0 (00%)	2 (100%)		
**Frequency washing hands**				
Less than 5 times (*n* = 31)	3 (9.7%)	28 (90.3%)	0.689	0.709
5 to 10 times (*n* = 97)	15 (15.5%)	82 (84.5%)		
More than 10 times (*n* = 52)	8 (15.4%)	44 (84.6%)		

*COVID-19* coronavirus disease 2019, *BSRS-5* 5-item Brief Symptom Rating Scale

^a^Chi square test (*χ*^2^)

^b^Cell of significance

**Table 6:** Relation between parameters and patients mental health degree (*n* = 180)

Parameters	BSRS-5 < 6 (***n*** = 26), no (%)	BSRS-5 ≥ 6 (***n*** = 154), no (%)	^**a**^ Test	***p*** value
**Your fear about**				
Infection (*n* = 26)	3 (11.5%)	23 (88.5%)	8.320	0.139
Family (*n* = 92)	16 (17.4%)	76 (82.6%)		
Quarantine (*n* = 6)	0.0 (00%)	6 (100%)		
Financial problems (*n* = 14)	1 (7.1%)	13 (92.9%)		
Medications (*n* = 11)	4 (36.4%)	7 (63.6%)		
Mixed fears (*n* = 31)	2 (6.5%)	29 (93.5%)		
**Are you at risk**				
No (*n* = 23)	6 (26.1%)	17 (73.9%)	7.036	0.030*
Yes (*n* = 97)	^b^8 (8.2%)	89 (91.8%)		
Maybe (*n* = 60)	12 (20%)	48 (80%)		
**Current disease activity**				
Decreased (*n* = 6)	1 (16.7%)	5 (83.3%)	1.211	0.546
Did not change (*n* = 131)	21 (16%)	110 (84%)		
Increased (*n* = 43)	4 (9.3%)	39 (90.7%)		
**Activity may increase due to**				
Fear (*n* = 92)	9 (9.8%)	83 (90.2%)	5.096	0.278
Postponed follow-up visits (*n* = 22)	4 (18.2%)	18 (81.8%)		
Reduced activity (*n* = 17)	5 (29.4%)	12 (70.6%)		
Drug shortage (*n* = 30)	5 (16.7%)	25 (83.3%)		
Drug incompliance (*n* = 19)	3 (15.8%)	16 (84.2%)		
**Contact physician**				
Less than usual (*n* = 80)	13 (16.2%)	67 (83.8%)	1.428	0.699
As usual (*n* = 44)	7 (15.9%)	37 (84.1%)		
More than usual (*n* = 5)	0.0 (00%)	5 (100%)		
I did not (*n* = 51)	6 (11.8%)	45 (88.2%)		
**Means of communication**				
Phone call (*n* = 29)	4 (13.8%)	25 (86.2%)	3.631	0.458
Clinic (*n* = 50)	6 (12%)	44 (88%)		
Internet (*n* = 63)	13 (20.6%)	50 (79.4%)		
Send someone (*n* = 2)	0.0 (00%)	2 (100%)		
I did not (*n* = 36)	3 (8.3%)	33 (91.7%)		
**Cause of communication**				
Disease activity or pain (*n* = 44)	4 (9.1%)	40 (90.9%)	5.575	0.350
COVID symptoms (*n* = 5)	0.0 (00%)	5 (100%)		
Drug shortage (*n* = 24)	5 (20.8%)	19 (79.2%)		
Medication advice (*n* = 51)	11 (21.6%)	40 (78.4%)		
Ask about COVID (*n* = 6)	1 (16.7%)	5 (83.3%)		
I did not (*n* = 50)	5 (10%)	45 (90%)		
**Feeling after communication**				
Feel more anxiety and fear (*n* = 14)	1 (7.1%)	13 (92.9%)	3.985	0.263
Feel the same (*n* = 75)	9 (12%)	66 (88%)		
Feel less anxiety and fear (*n* = 37)	9 (24.3%)	28 (75.7%)		
I did not (*n* = 54)	7 (13%)	47 (87%)		
**Fear of drugs** (*n* = 108)	20 (18.5%)	88 (81.5%)	3.626	0.057
**Stopped drugs** (*n* = 148)	24 (16.2%)	124 (83.8%)	Fisher	0.146
**Experienced drug shortage** (*n* = 123)	17 (13.8%)	106 (86.2%)	0.122	0.727
**Reduced doses of drugs** (*n* = 122)	18 (14.8%)	104 (85.2%)	0.029	0.864

*COVID-19* coronavirus disease 2019, *BSRS-5* 5-item Brief Symptom Rating Scale

^a^Chi square test (*χ*^2^)

^b^Cell of significance

*P value <0.05 is significant

Comparison between patients with rheumatoid arthritis (RA), systemic lupus erythematosus (SLE), and other rheumatic diseases regarding different parameters showed that there was a statistically significant difference [*p* ≤ 0.05] between them as regards age, sex, their belief that a healthy diet and exercises may help, frequency of going outdoors, BSRS-5 total score, being at risk, their feeling after communication, stopping drugs and experiencing drug shortages. RA patients had higher mean age and a higher percent of the male sex, they believe that a healthy diet and exercises can help, they go out only for necessities, they feel “maybe/not at risk”, they feel less/the same anxiety and fear after communicating with their physicians, they are less adherent to some drugs and experience more drug shortages. SLE patients had a higher percentage of the female sex, they believe a healthy diet and exercises “maybe/not help,” mostly “never go outdoors,” they feel “yes at risk,” feel more/the same anxiety, and fear after communication, and most do not stop drugs. Patients with other rheumatic diseases had higher mean BSRS-5 a total score and mostly “go outdoors freely any time,” feel “not at risk,” and experienced no drug shortage.

Also, comparing between patients with RA and SLE only, a statistically significant difference [*p* ≤ 0.05] was found among them as regards some parameters. RA patients had higher mean age, lower mean BSRS-5 total score, and a higher percentage of the male sex, “yes healthy diet and exercises help,” feel “not at all/quite a bit inferiority,” feel “maybe/not at risk,” feel less anxiety and fear after communication, and stopped drugs more often.

## Discussion

COVID-19 pandemic has affected people's lives in many different aspects. The fact that neither vaccine nor confirmed effective treatment has been developed has made the Coronavirus a huge stressor and a fearful threat to many individuals [23]. With COVID-19, the social isolation, quarantine, and loneliness in addition to fear of infection or death were the main factors impacting not only those infected with COVID but also the general population. The resulting fears, although considered a usual response to this major threatening event, when chronic and profound becomes harmful and may lead to psychiatric illness [24, 25]**.**

Health professionals, including doctors and nurses, being the most at risk, suffered impaired mental health [26, 27]. Special population groups who are considered more vulnerable to infection- old age, immune-compromised, and patients with chronic illnesses—frequently complained of considerable psychological problems [7]. The “double crush” of having a chronic illness and receiving immunosuppressive drugs makes patients with rheumatic diseases among these groups more vulnerable to infection and thus impaired mental health.

Most patients were non-smokers and the majority had no previous lung affection. However, smokers and those with lung affection did not seem to have a different attitude or behavior regarding being at a greater risk, most probably due to the fact that most patients with chronic diseases had comparable degrees of anxiety. When comparing the attitude and behavior towards COVID among the studied groups, no statistically significant difference was found between cases and control group regarding their attitude towards staying at home, the value of a healthy diet and exercises and washing hands; while there was a statistically significant difference between both groups regarding going outdoors, also, wearing masks and gloves outdoors, where patients seemed to be more self-protecting. Self-isolation in their room most of the time was mostly practiced by patients more than controls.

Previous studies have shown that mental health disorders can last longer than the epidemic itself [8, 9]. Individuals with chronic illnesses are even more exposed to psychological impairment [16, 17]. Mental health comparison among the studied groups using the BSRS showed that there was a statistically significant difference between both groups regarding “feeling angry/irritated,” “feeling inferior to others,” insomnia, and regarding the BSRS total score. Out of all patients, 85.6% were defined as a psychiatric case. Their belief of “being at risk” was associated with a higher degree of impaired mental health [BSRS-5 ≥ 6]. These distress reactions [feeling angry, irritable, and disturbed sleep] being more prominent in cases denote fear and panic. About 20% were “extremely” angry and agitated. Moreover, cases were feeling more inferior, which is most probably related to their chronic illness. Ironically, no significant difference was found regarding anxiety and depression between cases and controls denoting that the general population during the pandemic felt similar degrees of anxiety and depression. Fear of infection or death raises anxiety levels in healthy individuals as well, quarantine and social isolation increased depression in both groups similarly. Both patients and healthy feared mostly losing their families and beloved ones.

Regarding the attitude and behavior of the cases towards medications; the highest proportion of them answered that they have no fear of drugs, they did not reduce doses, nor did they stop any of their drugs. Those who stopped drugs may have had financial issues or difficulty finding medications. This is a good positive attitude consistent with the EULAR guidelines regarding medications, representing their awareness of the necessity of continuing their medications to avoid flares [28]. Moreover, many of them did not experience any increase in their disease activity, although many believed that fear itself may cause a flare. The most frequent cause to contact their physician was medication advice rather than consultation due to pain and disease activity. The increased usage of antimalarial drugs to treat COVID-19 patients caused a decline in their availability in pharmacies, highlighting the harm to patients caused by medications being purchased in Egypt without medical prescription [29]. For some patients, this might be an essential treatment especially for those with SLE, and many had to communicate with their physicians for substitutes. During the pandemic, most of the patients visited their physicians less than usual to minimize going outdoors to healthcare facilities; they mostly contacted them using telehealth.

Regarding the comparison between patients with RA, SLE, and other rheumatic diseases, RA patients had higher mean age and a higher percent of the male sex, while SLE patients were mostly younger females. Most RA patients believe that a healthy diet and exercise can help combat infection while SLE patients are less confident about that. RA patients only go out for necessities while most SLE patients never go outdoors as they felt that they are at risk more than other patients. They even feel more/the-same anxiety and fear after communicating with their physicians. Most of SLE patients are adherent to their drugs more than other patients. We conclude that although rheumatologists succeeded to convince their patients to stick to their medications, they failed to relieve their anxiety and fear which may be augmented by media, following numbers of those COVID-infected and dead. About 48% depended on social media as a source of information about COVID-19. Both RA patients and SLE patients experienced drug shortages. Most patients with other rheumatic diseases go outdoors more freely, they do not feel great that they are at a higher risk like SLE patients nor do they significantly complain of drug shortages as they are less dependent on anti-malarials unlike SLE patients.

Regarding their mental status RA patients had significantly lower mean BSRS-5 total score and felt less inferiority than SLE patients. No significant difference was found in patients with different diseases regarding anxiety and depression. Although cases were significantly different regarding anger and insomnia compared to controls, there were no differences comparing patients with different disease categories.

The limitations of our study were the inability to assess the same patients’ psychological status just before the hit of coronavirus, an unexpected event, which would have been more informative about the direct impact of COVID-19 pandemic isolated from the effect of their chronic illnesses which had been also accused to affect mental health [30, 31]. The finding that mental health status was more affected by their belief of “being at risk” of infection was a clear indicator of the impact of COVID-19 pandemic. The presented data are preliminary data that could be obtained during the quarantine period and we are planning for future studies to test behavioral changes on rheumatic patients that had suffered from COVID-19 infection. Another limitation is recruiting patients who use social media with no inclusion of patients not using it as illiterate patients and patients from low socioeconomic that are not familiar with social media.

## Conclusion

Patients with rheumatic diseases show comparable degrees of anxiety and depression to healthy individuals, but higher distress symptoms and panic in the form of anger, irritability, and insomnia. They have a significantly higher sense of inferiority and a higher total BSRS compared to controls. SLE patients show more adherence to their medications and stay mostly at home as a reflection of feeling more vulnerable. Moreover, they have higher degrees of psychological affection in the form of higher BSRS scores. Abandoning drug purchasing without medical prescription is necessary in Egypt to protect our patients from unnecessary drug shortages adding to their fear and anxiety. Mental health should be addressed in the same manner we deal with the infectious disease itself, being of no less importance. Mental health professionals, social workers, and support groups need to provide psychological support to vulnerable populations, including patients with rheumatic diseases. Rheumatologists should be aware of the need for psychiatric consultation for their patients whenever necessary.

## Supplementary Information

**Additional file 1.**

## Data Availability

Available upon request.

## Abbreviations

COVID-19: Coronavirus disease 2019
BSRS-5: Brief Symptom Rating Scale
SARS-CoV-2: Severe acute respiratory syndrome coronavirus 2
WHO: World Health Organization
ACR: American College of Rheumatology
IRB: Institutional review board
RA: Rheumatoid arthritis
SLE: Systemic lupus erythematosus

## References

[1] Lu H, Stratton CW, Tang Y-W (2020). Outbreak of pneumonia of unknown etiology in Wuhan, China: the mystery and the miracle. J Med Virol.

[2] Who.com. World Health Organization. Available from: https://time.com/5791661/who-coronavirus-pandemic-declaration. cited May, 24 2020

[3] Wu KK, Chan SK, Ma TM (2005). Posttraumatic stress, anxiety, and depression in survivors of severe acute respiratory syndrome [SARS]. J Trauma Stress.

[4] Chong M-Y, Wang W-C, Hsieh W-C, Lee C-Y, Chiu N-M, Yeh W-C (2004). Psychological impact of severe acute respiratory syndrome on health workers in a tertiary hospital. Br J Psychiatry.

[5] Goulia P, Mantas C, Dimitroula D, Mantis D, Hyphantis T (2010). General hospital staff worries, perceived sufficiency of information and associated psychological distress during the a/H1N1 influenza pandemic. BMC Infect Dis.

[6] Docea AO, Tsatsakis A, Albulescu D, Cristea O, Zlatian O, Vinceti M (2020). A new threat from an old enemy: re-emergence of coronavirus [review]. Int J Mol Med.

[7] Xiang Y-T, Yang Y, Li W, Zhang L, Zhang Q, Cheung T (2020). Timely mental health care for the 2019 novel coronavirus outbreak is urgently needed. Lancet Psychiatry.

[8] Reardon S (2015). Ebola's mental-health wounds linger in Africa. Nature.

[9] Shigemura J, Ursano RJ, Morganstein JC, Kurosawa M, Benedek DM (2020). Public responses to the novel 2019 coronavirus [2019-nCoV] in Japan: mental health consequences and target populations. Psychiatry Clin Neurosci.

[10] Malta M, Rimoin AW, Strathdee SA (2020). The coronavirus 2019-nCoV epidemic: is hindsight 20/20?. E Clinical Medicine.

[11] Cascella M, Rajnik M, Cuomo A, Dulebohn SC, Di Napoli R (2020). Features, evaluation and treatment coronavirus [COVID-19].

[12] Peeri NC, Shrestha N, Rahman MS. et al. (2020) The SARS, MERS and novel coronavirus (COVID-19) epidemics, the newest and biggest global health threats: what lessons have we learned? Int J Epidemiol 1;49(3):717–726. 10.1093/ije/dyaa033. 10.1093/ije/dyaa033 PMC7197734 32086938

[13] Ornell F, Schuch JB, Sordi AO, Kessler FHP (2020). “pandemic fear” and COVID-19: mental health burden and strategies. Rev Bras Psiquiatr.

[14] Buheji M, Buheji A (2020). Planning competency in the new Normal– employability competency in post- COVID-19 pandemic. Int J Hum Resour Stud.

[15] Li W, Yang Y, Liu Z-H, Zhao Y-J, Zhang Q, Zhang L, Cheung T, Xiang Y-T (2020). Progression of mental health services during the COVID-19 outbreak in China. Int J Biol.

[16] Applegate WB, Ouslander JG (2020). COVID-19 presents high risk to older persons. J Am Geriatr Soc.

[17] Dong XC, Li JM, Bai JY, Liu ZQ, Zhou PH, Gao L (2020). Epidemiological characteristics of confirmed COVID-19 cases in Tianjin. Zhonghua Liu Xing Bing Xue Za Zhi.

[18] Mikuls TR, Johnson SR, Fraenkel L, Arasaratnam RJ, Baden LR, Bermas BL, Saag KG (2020) American College of Rheumatology Guidance for the Management of Adult Patients with rheumatic disease during the COVID-19 pandemic. Arthritis Rheumatol. Available from. 10.1002/art.41301 10.1002/art.41301 32349183

[19] Wang C, Pan R, Wan X, Tan Y, Xu L, Ho CS (2020). Immediate psychological responses and associated factors during the initial stage of the 2019 coronavirus disease [COVID-19] epidemic among the general population in China. Int J Environ Res Public Health.

[20] Lee H, Han J, Kang J (2020. Available from:) Validity and reliability of the Korean anesthesia surrendering instrument. Res Square. 10.21203/rs.2.22075/v2 10.3390/ijerph17093065 PMC7246766 32354188

[21] Chen HC, Wu CH, Lee YJ (2005). Validity of the five-item brief symptom rating scale among subjects admitted for general health screening. J Formos Med Assoc.

[22] Lung FW, Lee MB (2008). The five-item brief-symptom rating scale as a suicide ideation screening instrument for psychiatric inpatients and community residents. BMC Psychiatry.

[23] Hawryluck L, Gold WL, Robinson S, Pogorski S, Galea S, Styra R (2004). SARS control and psychological effects of quarantine, Toronto, Canada. Emerg Infect Dis.

[24] Garcia R (2017). Neurobiology of fear and specific phobias. Learn Mem.

[25] Shin LM, Liberzon I (2010). The neurocircuitry of fear, stress, and anxiety disorders. Neuropsychopharmacology.

[26] Kang L, Li Y, Hu S, Chen M, Yang C, Yang BX (2020). The mental health of medical workers in Wuhan, China dealing with the 2019 novel coronavirus. Lancet Psychiatry.

[27] Huang JZ, Han MF, Luo TD, Ren AK, Zhou XP. (2020) [Mental health survey of medical staff in a tertiary infectious disease hospital for COVID-19]. Zhonghua Lao Dong Wei Sheng Zhi Ye Bing Za Zhi. 20;38(3):192–195. Chinese. 10.3760/cma.j.cn121094-20200219-00063. 10.3760/cma.j.cn121094-20200219-00063 32131151

[28] EULAR | EULAR Guidance for patients COVID-19 outbreak. Available from: https://www.eular.org/eular_guidance_for_patients_covid19_outbreak.cfm. Cited May 24, 2020

[29] Spinelli FR, Ceccarelli F, Di Franco M, Conti F (2020) Response to 'To consider or not antimalarials as a prophylactic intervention in the SARS-CoV-2 [Covid-19] pandemic’ by Parperis. Ann Rheum Dis:2020–217634. Available from:. 10.1136/annrheumdis-2020-2176 10.1136/annrheumdis-2020-217634 32366521

[30] Lwin MN, Serhal L, Holroyd C, Edwards CJ (2020). Rheumatoid arthritis: the impact of mental health on disease: a narrative review. Rheumatol Ther.

[31] Dregan A, Matcham F, Harber-Aschan L, Rayner L, Brailean A, Davis K, Hatch S, Pariante C, Armstrong D, Stewart R, Hotopf M (2019). Epidemiology (common mental disorders within chronic inflammatory disorders: a primary care database prospective investigation). Ann Rheum Dis.

