# Supplementary material for: Impact of coronavirus disease 2019 (COVID-19) pandemic on attitude, behavior, and mental health of patients with rheumatic diseases
Source: Egypt Rheumatol Rehabil. 2020 Nov 12;47(1):45. doi: 10.1186/s43166-020-00045-y (PMC7658909; doi:10.1186/s43166-020-00045-y)
Supplement: Supplementary file 1 — Additional file 1. [file 43166_2020_45_MOESM1_ESM.zip › CORONAVIRUS & Rheumatology patients2 - Google FormsR3.pdf]

CORONA VIRUS & RHEUMATIC PATIENTS

For patients with autoimmune rheumatological diseases (Rheumatoid arthritis, systemic lupus, scleroderma, ankylosing spondylitis, Sjogren's disease, poly/dermatomyositis or any other....)

We are concerned about the impact of CORONAVIRUS pandemic on your attitude, behavior and how you feel about. To what extent are you worried?

This will take about 5 to 10 minutes to fill.
We hope that this survey would provide any help to patients and rheumatologists.

* Required

Personal Information

1. Age? *

____

2. Sex? *

Mark only one oval.

- ( ) Male
- ( ) Female

3. Nationality? *

Mark only one oval.

- Egyptian
- Non-Egyptian

4. Where do you live?

Mark only one oval.

- In Egypt
- Outside Egypt

5. Do you smoke? *

Mark only one oval.

- Yes
- No

6. What is your rheumatological disease? *

____

7. Did your rheumatological disease affect your lungs? *

Mark only one oval.

- Yes
- No

8. Are you infected with CORONAVIRUS? *

Mark only one oval.

- Yes
- No

Information source about coronavirus

9. I most frequently get my knowledge about coronavirus from *

Mark only one oval.

- TV /Radio
- I search the internet
- Social media groups
- My own physician
- Friends and family

Attitude

10. Do you feel that you are at a higher risk than others? *

Mark only one oval.

- Yes
- No
- Maybe

11. Do you think that staying at home will protect you? *

Mark only one oval.

- Yes
- No
- Maybe

12. Do you think that wearing a mask will protect you? *

Mark only one oval.

- Yes
- No
- Maybe

13. Do you think that wearing gloves will protect you? *

Mark only one oval.

- Yes
- No
- Maybe

14. Do you think washing your hands with water and soap will protect you? *

Mark only one oval.

- Yes
- No
- Maybe

15. Do you think that a healthy diet and home exercises can help? *

Mark only one oval.

- Yes
- No
- Maybe

16. My disease activity during the Corona pandemic? *

Mark only one oval.

- Increased
- Did not change
- Decreased

17. Do you think your disease activity may increase mostly because of *

Mark only one oval.

- Anxiety and fear
- Drug shortage in pharmacies
- No being able to follow up with my physician regularly
- Not taking all drugs optimally being afraid of it
- Not moving alot and staying at home

Anxiety and fear

18. To what extent have you been feeling tense or keyed up (in the past week)? *

Mark only one oval.

- Not at all
- A little bit
- Moderately
- Quite a bit
- Extremely

19. To what extent have you been feeling blue (in the past week) *

Mark only one oval.

- Not at all
- a little bit
- moderately
- Quite a bit
- Extremely

20. To what extent did you feel annoyed or irritated (in the past week)? *

Mark only one oval.

- Not at all
- A little bit
- Moderately
- Quite a bit
- Extremely

21. To what extent do you feel inferior to others (in the past week)? *

Mark only one oval.

- Not at all
- A little bit
- Moderately
- Quite a bit
- Extremely

22. To what extent do you have trouble falling asleep (in the past week)? *

Mark only one oval.

- Not at all
- A little bit
- Moderately
- Quite a bit
- Extremely

23. The most you are afraid of or worried about is *

Check all that apply.

- [ ] To get the Coronavirus
- [ ] That my family or friends get the virus
- [ ] The idea of isolation and quarantine
- [ ] Financial problems
- [ ] Immunosuppressive rheumatology drugs

Behavior

24. How often do you go outdoors? *

Mark only one oval.

- Never
- Only for necessities
- I go out freely any time

25. Do you wear a mask if you go out? *

Mark only one oval.

- Always
- Most of the time
- Few times
- Never
- I don't go out

26. Do you wear gloves if you go out? *

Mark only one oval.

- Always
- Most of the times
- Few times
- Never
- I don't go out

27. Do you stay in your room away from your family members? *

Mark only one oval.

- All of the time
- Most of the time
- A bit of the time
- I move freely at home

28. About how many times do you wash your hands during the day? *

Mark only one oval.

- Less than 5 times
- 5 to 10 times
- More than 10 times

Contacting your physician

29. I contacted my doctor during the Corona pandemic *

Mark only one oval.

- More than usual
- As usual
- Less than usual

30. How do you contact your physician? *

Mark only one oval.

- Visited the clinic or hospital
- Call him by phone
- via the internet
- I sent him one of my relatives or friends

31. I contacted him/her most of the times due to: *

Mark only one oval.

- Disease activity or pain
- Fever, cough, dyspnea
- Drug shortage
- Asking about continuation of my drugs
- Asking about coronavirus

32. What do you feel after contacting your physician? *

Mark only one oval.

- I feel less anxiety and fear
- I feel the same
- I feel more anxiety and fear

Rheumatology drugs

33. Do you have fear taking your medications? *

Mark only one oval.

- Yes
- No

34. Did you stop taking any of your drugs? *

Mark only one oval.

- Yes
- No

35. Do you find difficulty finding some drugs like hydroxychloroquine? *

Mark only one oval.

- Yes
- No

36. Did you ask your physician to minimize the number of your drugs or your doses? *

Mark only one oval.

- Yes
- No

37. Did your physician instruct you how to deal with your drugs if you have any symptoms suggesting coronavirus *

Mark only one oval.

- Yes
- No

This content is neither created nor endorsed by Google.

Google Forms
