# Supplementary material for: Impact of coronavirus disease 2019 (COVID-19) pandemic on attitude, behavior, and mental health of patients with rheumatic diseases
Source: Egypt Rheumatol Rehabil. 2020 Nov 12;47(1):45. doi: 10.1186/s43166-020-00045-y (PMC7658909; doi:10.1186/s43166-020-00045-y)
Supplement: Supplementary file 1 — Additional file 1. [file 43166_2020_45_MOESM1_ESM.zip › renamed_1f45eR3.PDF]

تأثير وباء الكورونا على مرضى الروماتيزم المفصلي المناعى

السلام عليكم ورحمة الله وبركاته

هذا الاستبيان موجه لمرضى الروماتيزم المناعي مثل الروماتويد والزئبة الحمراء والتهاب العضلات المناعي المزمن ومرض بهجت والتيبس الفقاري والتهاب المفاصل الصدفي وتيبس الجلد وغيرها....

نحن مهتمون بمعرفة مدى تأثير وباء الكورونا على حياتك اليومية وإلى أي مدى انت خائف او قلق بشأن العلاج والمرض والإصابة بالفيروس... هل تعتقد انك معرض اكثر من غيرك ؟ هل تتواصل مع طبيبك الخاص؟ وكيف؟ هل شعرت بنشاط زائد للمرض ؟ هل تعاني من نقص الدواء ؟

يستغرق هذا الاستبيان ٥ الى ١٠ دقائق. نتمنى ان يكون مفيد للمرضى والأطباء في تعاملهم مع المرضى.

* Required

البيانات الشخصية

1. السن ؟ *

2. النوع ؟ *

Mark only one oval.

- ( ) ذكر
- ( ) أنثى

3. ما هي جنسيتك؟ *

Mark only one oval.

- ( ) مصري
- ( ) غير مصري

4. اين تسكن؟

Mark only one oval.

- ( ) في مصر
- ( ) خارج مصر

5. هل انت مدخن؟ *

Mark only one oval.

- ( ) نعم
- ( ) لا

6. ما هو المرض الروماتيزمي الذي تعاني منه؟ *

____

7. هل تأثر جهازك التنفسي والرئتين بسبب هذا المرض؟ *

Mark only one oval.

- ( ) نعم
- ( ) لا

8. هل انت مصاب بالكورونا ؟ *

Mark only one oval.

- ( ) نعم
- ( ) لا

مصادر المعلومات عن الكورونا

9. احصل على المعلومات بخصوص فيروس كورونا في اغلب الاوقات من *

Mark only one oval.

- التلفاز او الراديو
- ابحث في الانترنت
- صفحات التواصل الاجتماعي
- طبيبي الخاص
- أصدقائي وعائلتي

موقفك من / رأيك

10. هل تظن انك معرض للاصابه بفيروس الكورونا اكثر ممن حولك؟ *

Mark only one oval.

- نعم
- لا
- ربما

11. هل تظن ان الجلوس في المنزل قد يحميك؟ *

Mark only one oval.

- نعم
- لا
- ربما

12. هل تظن ان ارتداء ماسك قد يحميك؟ *

Mark only one oval.

- نعم
- لا
- ربما

13. هل تظن ان ارتداء القفازات قد يحميك؟ *

Mark only one oval.

- نعم
- لا
- ربما

14. هل تظن ان غسيل الايدي بالماء والصابون قد يحميك؟ *

Mark only one oval.

- نعم
- لا
- ربما

15. هل تظن ان الاكل الصحي وممارسة الرياضة المنزلية قد يحميك؟ *

Mark only one oval.

- نعم
- لا
- ربما

16. نشاط المرض الروماتيزمي عندي منذ بداية وباء الكورونا؟ *

Mark only one oval.

- ( ) زاد
- ( ) لم يتغير
- ( ) قل

17. هل تظن ان نشاط المرض قد يزيد عندك غالبا بسبب؟ *

Mark only one oval.

- ( ) التوتر والخوف والقلق
- ( ) نقص الدواء في الصيدليات
- ( ) عدم المتابعة بشكل منتظم مع الطبيب بسبب الحظر
- ( ) عدم أخذ العلاج كاملا خوفا منه
- ( ) قلة الحركة والبقاء في المنزل

القلق والخوف

18. الى اي مدى تشعر بالتوتر والقلق *

Mark only one oval.

- ( ) لا على الإطلاق
- ( ) قليلا
- ( ) متوسط
- ( ) اعلى من المتوسط
- ( ) إلى ابعد حد

19. الى اي مدى تشعر بالإكتئاب *

Mark only one oval.

- لا على الإطلاق
- قليلا
- متوسط
- أعلى من المتوسط
- إلى ابعد حد

20. الى اي مدى تشعر بالإنزعاج والغضب بسهوله

Mark only one oval.

- لا على الإطلاق
- قليلا
- متوسط
- أعلى من المتوسط
- إلى أبعد حد

21. الى اي مدى تشعر بأنك أقل من الآخرين *

Mark only one oval.

- لا على الإطلاق
- قليلا
- متوسط
- أعلى من المتوسط
- إلى أبعد حد

22. الى اي مدى تشعر بالأرق وتجد صعوبة في الخلود إلى النوم *

Mark only one oval.

- ( ) لا على الإطلاق
- ( ) قليلا
- ( ) متوسط
- ( ) أعلى من المتوسط
- ( ) إلى أبعد حد

23. أكثر ما يثير خوفك وقلقك؟ *

Check all that apply.

- [ ] اخاف على صحتي من الاصابه بالفيروس
- [ ] اخاف على عائلتي من الاصابة بالفيروس
- [ ] أخاف من فكرة الحظر والحجر الصحي
- [ ] أخاف من أزمات مادية
- [ ] اخاف من علاج الروماتيزم

السلوك

24. الخروج من المنزل ؟ *

Mark only one oval.

- ( ) لا اخرج نهائيا
- ( ) اخرج احيانا للضروريات فقط
- ( ) اخرج بحرية في اي وقت

25. هل ترتدي ماسك عند الخروج؟ *

Mark only one oval.

- دائما
- معظم الوقت
- القليل من الوقت
- لا أرتديه نهائيا
- لا أخرج

26. هل ترتدي قفازات عند الخروج؟ *

Mark only one oval.

- دائما
- معظم الوقت
- القليل من الوقت
- لا ارتديه نهائيا
- لا أخرج

27. هل تعزل نفسك في غرفتك بعيدا عن افراد الاسرة ؟ *

Mark only one oval.

- كل الوقت
- الكثير من الوقت
- القليل من الوقت
- اتحرك بحرية في البيت

28. كم تقريبا عدد المرات التي تغسل يدك بالماء والصابون في اليوم؟ *

Mark only one oval.

- اقل من ٥ مرات
- من ٥ الى ١٠ مرات
- اكثر من ١٠ مرات

الذهاب الى الطبيب

29. تواصلت مع طبيبك الخاص خلال فترة الوباء؟ *

Mark only one oval.

- أكثر من المعتاد
- عدد المرات المعتاده
- اقل من المعتاد
- لم اتواصل خلال فترة الوباء

30. وسيلة التواصل مع الطبيب كانت في أغلب المرات؟ *

Mark only one oval.

- ذهبت الى الطبيب (العياده او المستشفى)
- تواصلت معاه تليفونا
- تواصلت معه عبر الانترنت
- ارسلت احد اقاربي او أصدقائي اليه
- لم اتواصل

31. اسباب التواصل؟ *

Mark only one oval.

- زيادة نشاط المرض
- ظهور حراره او كحه او ضيق تنفس
- نقص في الدواء في الصيدليات
- الاستفسار عن استمرارية علاج الروماتيزم
- الاستفسار عن اي شئ يخص فيروس الكورونا
- لم اتواصل

32. ماذا تشعر بعد استشارتك للطبيب عن اي شئ يخص الكورونا؟ *

Mark only one oval.

- أشعر بالطمأنينة
- لا يتغير احساسي ناحية الكورونا
- أشعر بالمزيد من القلق
- لم اتواصل

العلاج

33. هل عندك مخاوف من اخد العلاج *

Mark only one oval.

- نعم
- لا

34. هل توقفت عن أخد العلاج *

Mark only one oval.

- [ ] نعم
- [ ] لا

35. هل تجد صعوبة في العثور على بعض الادوية مثل الهيدروكين/بلاكينيل *

Mark only one oval.

- [ ] نعم
- [ ] لا

36. هل طلبت من الطبيب تقليل جرعات العلاج او الاستغناء عن بعضها *

Mark only one oval.

- [ ] نعم
- [ ] لا

37. هل نصحك الطبيب عن كيفية التعامل مع العلاج في حالة ظهور اي اعراض تنفسيه؟ *

Mark only one oval.

- [ ] نعم
- [ ] لا

This content is neither created nor endorsed by Google.

Google Forms
